# Filter paper collection of *Plasmodium falciparum* mRNA for detecting low-density gametocytes

**DOI:** 10.1186/1475-2875-11-266

**Published:** 2012-08-08

**Authors:** Sophie Jones, Colin J Sutherland, Cornelus Hermsen, Theo Arens, Karina Teelen, Rachel Hallett, Patrick Corran, Marga van der Vegte-Bolmer, Robert Sauerwein, Chris J Drakeley, Teun Bousema

**Affiliations:** 1 Department of Immunology & Infection; Faculty of Infectious and Tropical Diseases, London School of Hygiene and Tropical Medicine, London, UK; 2 Department of Medical Microbiology, Radboud University Nijmegen Medical Centre, Nijmegen, The Netherlands

**Keywords:** Reverse-transcription polymerase chain reaction (RT-PCR), Quantitative nucleic acid sequence-based amplification (QT-NASBA), Sub-microscopic, Gametocyte, Detection, Elimination, Transmission, Ribonucleic acid (RNA), Filter paper

## Abstract

**Background:**

Accurate sampling of sub-microscopic gametocytes is necessary for epidemiological studies to identify the infectious reservoir of *Plasmodium falciparum*. Detection of gametocyte mRNA achieves sensitive detection, but requires careful handling of samples. Filter papers can be used for collecting RNA samples, but rigorous testing of their capacity to withstand adverse storage conditions has not been fully explored.

**Methods:**

Three gametocyte dilutions: 10/μL, 1.0/μL and 0.1/μL were spotted onto Whatman™ 903 Protein Saver Cards, FTA Classic Cards and 3MM filter papers that were stored under frozen, cold chain or tropical conditions for up to 13 weeks . RNA was extracted, then detected by quantitative nucleic acid sequence-based amplification (QT-NASBA) and reverse-transcriptase PCR (RT-PCR).

**Results:**

Successful gametocyte detection was more frequently observed from the Whatman 903 Protein Saver Card compared to the Whatman FTA Classic Card, by both techniques (p < 0.0001). When papers were stored at higher temperatures, a loss in sensitivity was experienced for the FTA Classic Card but not the 903 Protein Saver Card or Whatman 3MM filter paper. The sensitivity of gametocyte detection was decreased when papers were stored at high humidity.

**Conclusions:**

This study indicates the Whatman 903 Protein Saver Card is better for Pfs25 mRNA sampling compared to the Whatman FTA Classic Card, and that the Whatman 3MM filter paper may prove to be a satisfactory cheaper option for Pfs25 mRNA sampling. When appropriately dried, filter papers provide a useful approach to Pfs25 mRNA sampling, especially in settings where storage in RNA-protecting buffer is not possible.

## Background

The recent decline in the burden of malaria has placed malaria elimination and eradication back on the agenda of the international research community and health policy makers [1-4]. As a consequence, vaccines and anti-malarial drugs that specifically target the transmission stages of malaria parasites, gametocytes, are a high priority [1,2]. These interventions aim to reduce the infectious reservoir of malaria: the number of individuals capable of infecting mosquitoes. Infectiousness of humans to mosquitoes depends on the presence of mature gametocytes in the peripheral blood, and sensitive detection of gametocytes is therefore paramount. In the last decade, it has become evident that detection of gametocytes by microscopy is insufficiently sensitive to assess potential infectivity. Gametocyte densities below the microscopic threshold for gametocyte detection (~ 5 gametocytes/μL) frequently result in mosquito infection [5-8]. Molecular detection of low levels of gametocyte specific mRNA enables identification of submicroscopic gametocyte carriers, and has revealed gametocyte prevalence to be four to ten times higher than estimated by microscopy [9-20].

However, whilst mRNA-based gametocyte detection has aided epidemiological research concerned with low density *Plasmodium falciparum* gametocytes, the labile nature of RNA and the ubiquitous presence of RNAses pose challenges to sampling under field conditions, where high heat and humidity may lead to mRNA degradation [21]. Optimal handling of RNA samples, eg, stabilizing in buffer, shipping on dry ice, and storing at −80°C, allows maintenance of integrity of nucleic acids but restricts sampling to well-equipped laboratories. To facilitate RNA sampling and storage in settings with limited resources a filter paper-based approach has been proposed [22]. Filter papers have been routinely used for reliable collection and storage of whole blood samples for both antibody and DNA recovery [23,24]. The value of filter-paper matrices for malaria mRNA collection remains to be confirmed, in particular for detecting low-density infections under field conditions. In this study, the suitability of three different filter papers that are recommended for DNA or RNA storage has been determined for the mRNA-based detection of low-density gametocyte concentrations. Samples were stored on two filter papers under different conditions, ranging from those available in well-equipped laboratories to humid and hot tropical conditions, whereas the third paper was included in a smaller subset of conditions Two commonly used RNA extraction protocols were compared and gametocytes were detected by both reverse-transcriptase PCR (RT-PCR) and quantitative nucleic acid sequence based amplification (QT-NASBA).

## Methods

**Figure 1:**
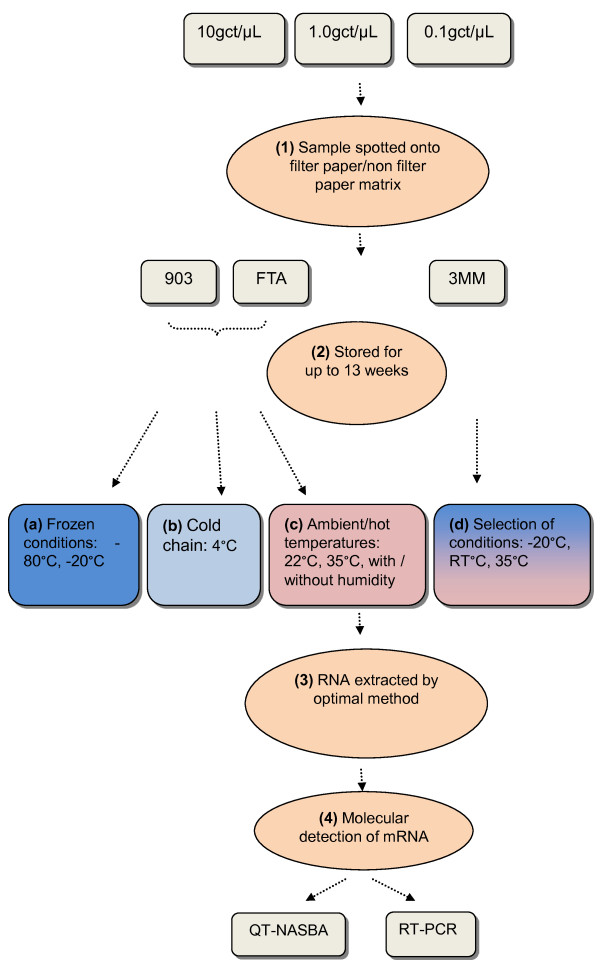
Overview of experimental procedure.

Figure 1 depicts an overview of the methodology. *Plasmodium falciparum* gametocytes were cultured as previously described [25,26], quantified in counting chambers by two independent microscopists and diluted to densities of 10, 1.0 and 0.1 gametocytes per μL in parasite negative European whole blood. These concentrations were chosen because they span the microscopic threshold for gametocyte detection [27] but may still be detectable by molecular methods [28]. Large blood spots of fifty μL of the different dilutions were aliquoted in five replicates per filter paper, incubation condition and time point, and allowed to dry overnight before being sealed into plastic bags with a silica desiccant sachet. To evaluate the impact of sub-optimal drying on mRNA recovery, after aliquoting the sample some papers were immediately sealed into bags without air drying overnight, and were stored without desiccant in containers with ~80 % relative humidity. Two sets of papers were handled this way and are referred to as the conditions ‘with humidity’ in Figure 1.

Three filter paper types were evaluated: the Whatman 903 Protein Saver Card, Whatman FTA Classic Card and the Whatman 3MM filter paper (GE Healthcare Ltd., New Jersey, USA). The FTA Classic Card was included as it is impregnated with chemicals that lyse cells, denature proteins and protect nucleic acids from nucleases, oxidative and UV damage. The 903 Protein Saver Card, has no stabilizing properties but has previously been shown to be suitable for RNA collection [22,29]. The 3MM plain filter paper has no nucleic acid stabilizing properties, was not expected to perform well and was therefore evaluated under a smaller subset of conditions [30]. Storage conditions were considered to replicate likely conditions that may be encountered at field sites for sampling and during transportation back to the laboratory. Frozen and cold chain incubation temperatures were: -80°C, -20°C and 4°C. To represent laboratories without access to any cold storage, ambient/room temperature (an average of 22°C, but with a range from 20-24°C) and hot tropical temperatures (35°C, in an incubator) conditions were included. To mimic sampling under humid conditions, papers were also stored at 22°C and at 35°C in a wet box with a hygrometer, reaching ~ 80 % relative humidity. Filter papers were incubated for one week under a ‘transportation/shipping’ temperature, then moved to a second ‘storage’ temperature, where replicates were kept for an additional four weeks or twelve weeks before RNA extraction.

As non- filter paper controls, 50 μL aliquots of the three gametocyte densities and parasite negative diluent blood were stored in RNA stabilizing guanidine isothiocyanate buffer [31], at −80°C. A total of twenty replicates of gametocyte negative human blood was blotted onto 903 Protein Saver Cards; twenty onto the FTA Classic Card and ten onto the 3MM papers. These negative controls were stored at −20°C or 35°C with silica gel desiccant before extraction and analysis.

### RNA extraction

To determine optimum extraction methods, a small-scale comparison extracting total RNA with the RNeasy Mini Blood kit (QIAGEN Ltd, Crawley, UK) and a guanidine based extraction protocol [22,31] was conducted. Replicates of all three gametocyte densities were spotted onto the 903 Protein Saver Card (n = 21) and the FTA Classic Card (n = 21) which were air dried overnight then stored at −80°C or −20°C for twenty-four hours. RNA was extracted by both methods and sensitivity was determined by QT-NASBA, and measured in terms of the number of positive results. RNeasy extraction from filter papers was performed as described elsewhere, following an initial homogenization step where entire 50 μL blood spots were excised from filter papers then rocked horizontally in individual eppendorfs at 150 rpm for one hour with 500 μL of RNeasy Lysis Buffer (QIAGEN Ltd, Crawley, UK) plus β-mercaptoethanol (0.14 M final concentration). Following this, samples were centrifuged through QIAshredder columns (QIAGEN Ltd, Crawley, UK) as described elsewhere [22].

For guanidine-based extraction, filter papers were similarly homogenized in 700 μL of guanidine isothiocyanate L6 buffer (8.3 M GuSCN, 82 mM Tris–HCl pH 6.4, 36mMM EDTA pH 8 and 2 % Triton-X-100) and rocked for 1.5 hours at 150 rpm. After this, the supernatant was kept and an additional 700 μL of guanidine isothiocyanate L6 buffer was added to the filter paper that was rocked for thirty more minutes at 150 rpm. Then the supernatants were pooled and RNA extraction performed as described [31]. Non filter paper controls were gametocyte concentrations of 10, 1.0 and 0.1 gametocytes per μL in parasite negative European whole blood that was stored immediately in L6 buffer, and were extracted according to protocol from this media [31].

### Gametocyte detection

Gametocyte specific Pfs25 mRNA transcripts were detected in extracted material using quantitative nucleic acid sequence based amplification (QT-NASBA) and reverse transcription polymerase chain reaction (RT-PCR). QT-NASBA was conducted as previously described using the NucliSENS Easy Q Basic Kit, Version 2 (BioMérieux Benelux B.V, Boxtel, The Netherlands), see Table S1in the additional files. for primers and molecular beacon [32]. Prior to RT-PCR, potentially contaminating DNA was digested with DNA-free^TM^ (Applied Biosystems, Warrington, UK). Twenty μL reactions were conducted according to the manufacturer’s protocol with the following amendments: RNA sample was added to 2 μL of rDNase I, 2 μL of 10X DNase I buffer, and inactivated with 4 μL of DNase inactivation reagent. Nested RT-PCR was conducted using a previously published protocol with novel primers, described in Table S1 in the additional files and ammended cycling conditions [22,33]. Reactions were set up using lyophilized Illustra Ready to Go RT-PCR beads (Illustra, GE Healthcare UK Ltd, Buckinghamshire, UK) and PCR cycling was conducted as described below. To generate cDNA, reactions were held at 42°C for 30 minutes, followed by 95°C for five minutes. Primary PCR conditions were 94°C for two minutes, then 45 cycles of: 94°C for thirty seconds, 52°C for one minute and 68°C for 2.5 minutes.. Nested PCR was performed using two μL of primary PCR product and the same conditions as before, but with thirty cycles instead. The PCR product was analysed on a 1.5 % agarose gel and produced a band of ~124 base pairs.

### Data analysis

Statistical comparisons of the filter paper types and detection methods were performed using statistical software STATA Version 11 (Statacorp, Texas, USA). Comparisons of filter paper or extraction performance were done by Pearson’s χ2 or trend test. Where appropriate, multivariate logistic regression models were used to allow for the effect of gametocyte concentrations when comparing the effect of storage temperature by filter paper type and to allow for the effects of gametocyte concentrations and storage temperature when comparing the performance of filter papers. GraphPadPrism Version 5 (GraphPad Software Inc., La Jolla, USA) was used for graphical presentation.

## Results

### Selection of an RNA extraction method

In a pilot experiment RNA extraction from bloodspots on desiccated 903 Protein Saver Cards stored at −80°C or −20°C was found to be similar using guanidine-based extraction (18/21 successful amplifications) or RNeasy based extraction (19/21 successful amplifications), with no significant difference found between the two (p = 0.635), Table 1 RNA extraction from the FTA Classic Card was less efficient by RNeasy (12/21 successful amplifications) compared to guanidine-based extraction (17/21 successful amplifications), although this difference was not significant (p = 0.095), Table 1. Therefore, all subsequent experiments were continued with guanidine-based extraction, which is less laborious and less expensive.

**Table 1:** RNeasy Mini Blood kit (QIAGEN Ltd, Crawley, UK) and Boom RNA extraction technique comparison, determined by QT-NASBA

	**−80 °C**			**−20 °C**			**−80 °C**			**−20 °C**		
	**903 protein saver**						**FTA TM Classic Card**					
Extraction type	10	1	0.1	10	1	0.1	10	1	0.1	10	1	0.1
Boom	(5/5)	(5/5)	(4/5)	(2/2)	(1/2)	(1/2)	(5/5)	(5/5)	(1/5)	(2/2)	(2/2)	(2/2)
RNeasy	(5/5)	(5/5)	(4/5)	(2/2)	(2/2)	(1/2)	(5/5)	(3/5)	(1/5)	(2/2)	(1/2)	(0/2)

### Positive and negative controls

For non-filter paper controls when gametocyte dilutions were stored at −80°C in protective guanidine buffer, all of the samples with 10 gametocytes /μL or one gametocyte /μL were detected by QT-NASBA, but six out of eight were detected for both dilutions by RT-PCR. For the 0.1 gametocytes/μL samples five of eight were successfully detected by QT-NASBA, and three of eight for RT-PCR (Table 2). None of the negative European donor blood showed RNA amplification by either QT-NASBA or RT-PCR (0/23).

**Table 2:** Detection comparison for QT-NASBA and RT-PCR for whole blood stored directly in guanidine RNA preservation buffer at −80 °C

	**Gametocyte density/μl**			
	**10**	**1**	**0.1**	**negative**
QT NASBA	100 (8/8)	100 (8/8)	62.5 (5/8)	0 (0/4)
RT-PCR	75 (6/8)	75 (6/8)	37.5 (3/8)	0 (0/4)

### Storage duration

There was no evidence for a difference in the proportion of successful amplifications between samples stored in their second condition for four weeks compared to those stored for 12 weeks by either QT-NASBA (regression analysis adjusting for gametocyte concentration and storage condition, p = 0.89) or RT-PCR (regression analysis adjusting for gametocyte concentration and storage condition, p = 0.49) (Tables  S1 and S2 in the additional files). Therefore, data from the two time-points was combined for the comparison of storage conditions for subsequent analysis.

### Detection of gametocytes from filter papers

#### Detection of gametocytes from filter papers stored at −80°C or −20°C

**Figure 2:**
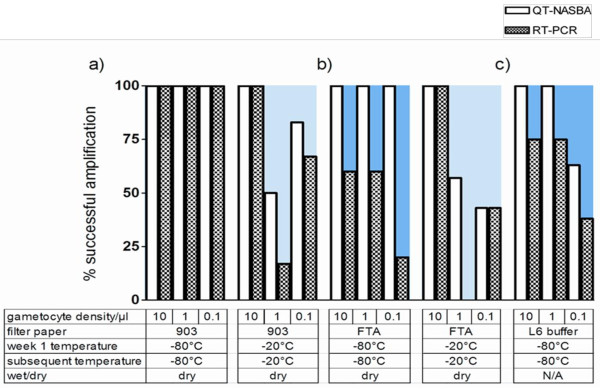
**Effect of frozen storage on gametocyte mRNA preserved on filter papers.** Percentage of mRNA amplification in 10, 1.0 and 0.1 gametocyte/μl dilutions, determined by QT-NASBA (open bars) and RT-PCR (hatched bars), when stored on filter paper. (**a**) shows the Whatman 903 Protein Saver filter paper, (**b**) the Whatman FTA Classic Card, and (**c**) sample stored in guanidine isothiocyanate buffer, when stored under frozen temperatures for up to thirteen weeks. The background blue to pink colour gradient represents the severity of the incubation condition (from cold to warm, respectively). Raw data is shown in Tables  S2 and S3, additional files.

All gametocyte concentrations that were blotted onto the 903 Protein Saver Cards and stored with desiccant at −80°C were successfully detected by QT-NASBA and RT-PCR (Figure 2a, Tables  S1 and S2 in the additional files). All gametocyte concentrations that were blotted onto the FTA Classic Cards and stored with desiccant at −80°C were successfully detected by QT-NASBA but not by RT-PCR (Figure 2a, p = 0.007).

**Figure 3:**
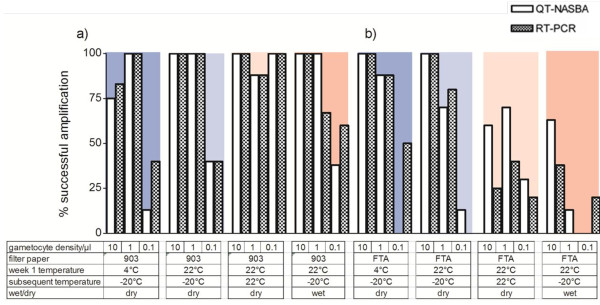
**Effect of cold chain storage on gametocyte mRNA preserved on filter papers.** Percentage of mRNA amplification in 10, 1.0 and 0.1 gametocyte/μl dilutions, determined by QT-NASBA (open bars) and RT-PCR (hatched bars), when stored on (**a**) the Whatman 903 Protein Saver filter paper and (**b**) the Whatman FTA Classic Card, under cold chain conditions with variable humidity for up to thirteen weeks. The background blue to pink colour gradient represents the severity of the incubation condition (from cold to warm, respectively).

**Figure 4:**
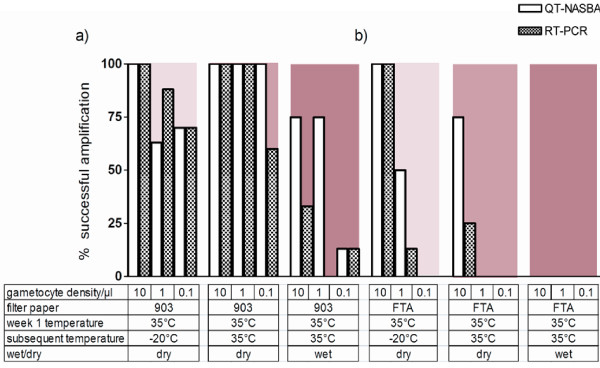
**Effect of tropical condition storage on gametocyte mRNA preserved on filter papers.** Percentage of mRNA amplification in 10, 1.0 and 0.1 gametocyte/μl dilutions, determined by QT-NASBA (open bars) and RT-PCR (hatched bars), when stored on (**a**) the Whatman 903 Protein Saver filter paper and (**b**) the Whatman FTA Classic Card, under tropical conditions with variable humidity for up to thirteen weeks. The background blue to pink colour gradient represents the severity of the incubation condition (from cold to warm, respectively).

Detection of gametocytes from filter papers stored at increasing temperatures The proportion of cards that gave successful amplification for all gametocyte dilutions was unaffected by increasing storage temperature if using the 903 Protein Saver Card (Figures 3–4, Tables  S2 and S3 in the additional files; (test for trend adjusting for gametocyte concentration, p = 0.18).

In contrast, there was evident loss in sensitivity when samples on FTA Classic Cards were stored at a higher temperature. Successful detection by QT-NASBA of gametocytes blotted on FTA Classic Cards was achieved for 100 % (9/9), 63.2 % (12/19), 53.3 % (16/30) and 31.6 % (6/19) of the samples that were continuously stored with desiccant at −80°C, -20°C, 22°C and 35°C, respectively (test for trend, p = 0.002). Similarly, successful detection by RT-PCR of gametocytes blotted on FTA Classic Cards was achieved for 46.7 % (7/15), 50.0 % (5/10), 27.8 % (5/18) and 10.5 % (2/19) of the samples that were continuously stored with desiccant at −80°C, -20°C, 22°C and 35°C, respectively (test for trend adjusting for gametocyte concentration, p = 0.02).

The use of RT-PCR for gametocyte detection led to similar conclusions about the effect of increasing storage temperature on the detectability of gametocytes from 903 Protein Saver Cards (test for trend adjusting for gametocyte concentration, p = 0.83) or FTA Classic Cards (test for trend adjusting for gametocyte concentration, p = 0.01).

#### Detection of gametocytes from filter papers stored at changing temperatures

Compared to 903 Protein Saver Cards that were continuously stored at −20°C, the chance of detecting gametocytes by QT-NASBA from 903 Protein Saver Cards that were first stored at higher temperatures (4°C, 22°C or 35°C) was not reduced (test for trend, p = 0.41). In contrast, FTA Classic Cards that were first stored at higher temperatures (4°C, 22°C or 35°C) were less frequently successfully amplified by QT-NASBA (test for trend adjusting for gametocyte concentration, p = 0.09). The use of RT-PCR for gametocyte detection resulted in similar conclusions about changing storage temperature and a loss in the detectability of gametocytes from 903 Protein Saver Cards (test for trend adjusting for gametocyte concentration, p = 1.00) and FTA Classic Cards (test for trend adjusting for gametocyte concentration, p = 0.09).

#### Detection of gametocytes from filter papers stored under humid conditions

A subset of filter papers were examined in humid and dry conditions; these samples were stored at 22°C for one week and subsequently shifted to −20°C (Figure 3) or were stored at 35°C for an additional 12 weeks (Figure 4). For the first condition, 22°C for one week followed by −20°C for the next 12 weeks , there was no reduction in the proportion of successfully amplified samples from 903 Protein Saver Card by QT-NASBA (regression analysis adjusting for gametocyte concentration, p = 0.87). However, there was a clear reduction in the proportion of successfully amplified samples from FTA Classic Cards by QT-NASBA (regression analysis adjusting for gametocyte concentration, p = 0.049). Comparisons between humid and dry conditions for these storage temperatures gave similar results when RT-PCR was used for gametocyte detection from 903 Protein Saver Cards (regression analysis adjusting for gametocyte concentration, p = 1.00) or FTA Classic Cards (regression analysis adjusting for gametocyte concentration, p = 0.009).

When stored at 35°C, there was a clear reduction in the proportion of successfully amplified samples from 903 Protein Saver Card by QT-NASBA (regression analysis adjusting for gametocyte concentration, p < 0.001). For FTA Classic Cards, too few samples gave successful amplification for statistical analysis; none of the samples stored under wet conditions at 35°C were successfully detected. Comparisons between humid and dry conditions for these storage temperatures gave similar results when RT-PCR was used for gametocyte detection.

#### Detection of gametocytes from Whatman 3MM filter papers

**Figure 5:**
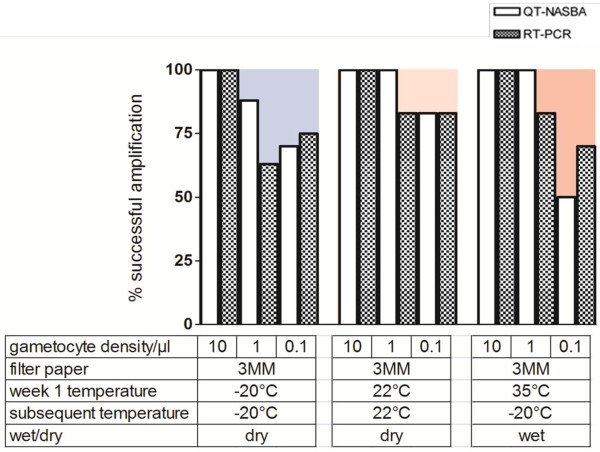
**Performance of Whatman 3MM as a storage media for gametocyte mRNA.** Percentage of mRNA amplification in 10, 1.0 and 0.1 gametocyte/μl dilutions, determined by QT-NASBA (open bars) and RT-PCR (hatched bars), when stored on the Whatman 3MM filter paper, incubated under different conditions for up to thirteen weeks. The background blue to pink colour gradient represents the severity of the incubation condition (from cold to warm, respectively).

Whatman 3MM papers were not expected to be a suitable media for mRNA storage, so were incubated under fewer conditions (Figure 5, Tables  S2 and S3, additional files). These samples were tested under three conditions: at −20°C for thirteen weeks, at 22°C for thirteen weeks and at 35°C for one week followed by −20°C storage for the next twelve weeks. When continuously stored at −20°C, there was no difference between the proportion of successfully detected samples for all gametocyte dilutions by QT-NASBA between 3MM and 903 Protein Saver Cards (regression analysis adjusting for gametocyte concentration = 0.34) or FTA Classic Cards (regression analysis adjusting for gametocyte concentration, p = 0.10). When stored continuously at 22°C, 3MM filter papers performed as well as 903 Protein Saver Cards (regression analysis adjusting for gametocyte concentration,p = 0.95) but better than FTA Classic Cards (regression analysis adjusting for gametocyte concentration,p = 0.01). When stored at 35°C for one week followed by −20°C for the next twelve weeks, 3MM filter papers performed as well as 903 Protein Saver Cards (regression analysis adjusting for gametocyte concentration, p = 0.75) but better than FTA Classic Cards (regression analysis adjusting for gametocyte concentration, p = 0.01). When RT-PCR was used, similar results were obtained.

## Discussion

This study was designed to investigate optimal collection and storage conditions to enable reliable detection of *P. falciparum* gametocyte densities found in natural infections. Filter papers were found to be a suitable media for Pfs25 mRNA detection from low gametocyte concentrations after storage for up to thirteen weeks. Furthermore, it was found that gametocyte Pfs25 mRNA stored on 903 Protein Saver Cards is remarkably stable at temperatures higher than −80°C.

Unlike the general detection of malaria parasites, which is based on amplification of *Plasmodium* nuclear or mitochondrial DNA, the specific detection of gametocytes depends on mRNA. Reliable sampling for gametocytes currently depends on RNA extraction in the field [15,19] or storage of samples in stabilizing buffer at −80°C in order to protect the integrity of mRNA, which is much more labile than DNA. The main aims of this study were to determine which filter papers were most suitable for storage of gametocyte mRNA in dried whole blood samples, and to explore flexibility of storage conditions and whether less stringent conditions are permissible without affecting estimates of gametocyte prevalence. This study builds upon similar research evaluating RNA detection from filter paper [22] but provides more rigorous testing with a larger number of replicates in diverse incubation conditions including both high and low humidity, which is of particular relevance for sampling in the tropics. QT-NASBA reliably detected gametocyte densities of ten or one gametocyte/μL when immediately stored in RNA-stabilizing buffer at −80°C and was found to be more sensitive than RT-PCR for detecting low gametocyte concentrations. The higher sensitivity of QT-NASBA is unlikely to be explained by amplification of ssDNA [34]; but we do consider it plausible that the DNAse digestion step may have negatively influenced the sensitivity of RT-PCR. It is also important to note that we compared QT-NASBA with RT-PCR as it was previously used for Pfs25 mRNA detection [22,33]; more sensitive quantitative RT-PCR assays may become available that make this assay comparable in sensitivity to QT-NASBA. Under optimal conditions, storage in RNA-stabilizing buffer at −80°C, it was observed that not all samples with 0.1 gametocyte/μLwere detected by QT-NASBA. This may reflect variability in extraction efficiency or RNA amplification (Churcher *et al.* in preparation) or an inevitable consequence of very low pathogen concentrations and sample volumes (an average of five gametocytes per 50 μL blood sample blotted on the paper). The use of an extraction control could address uncertainties on extraction efficiency between samples but was not used in this study or in other studies using Pfs25 mRNA RT-PCR or NASBA [5,6,13,15,29,32,35-37].

Detection of gametocytes from filter papers stored at −80°C succeeded with a similar, but slightly higher frequency compared to detection of gametocytes from samples stored directly in RNA stabilizing buffer. The unexpected improved detection from filter paper samples has been previously acknowledged in similar studies [34] where it has been suggested the drying step enhances blood cell lysis, leading to a higher probability of detection. It is currently unclear whether this apparent superiority of filter papers over RNA stabilizing buffer is specific to Pfs25 mRNA and if other gametocyte mRNA targets are less well stored or recovered using filter papers.

The successful detection of gametocytes from filter papers stored at −80°C formed a useful starting point for analyses that aimed to determine the suitability of filter papers for storage under less favourable conditions. No evidence was found that increasing storage temperature resulted in a lower success rates for gametocyte detection from 903 Protein Saver Cards. Contrary to this, gametocyte detection from the FTA Classic Cards was most efficient when stored at −80°C, lower when stored at −20°C or 22°C and lowest when stored at 35°C. FTA Classic Cards were also more susceptible to changes in storage temperature with short-term storage at temperatures higher than −20°C resulting in a loss in detectability of gametocytes from these cards. Qualitative gametocyte detection from 903 Protein Saver Cards was not affected by different storage temperatures.

Incomplete drying of filter papers resulted in a loss in efficiency of gametocyte detection. This effect was most apparent at the highest temperature tested (35°C) and FTA Classic Cards appeared most susceptible to this detrimental effect of moisture on gametocyte detection. This loss in sensitivity could be a result of RNA degradation and/or incomplete sample elution from insufficiently dried filter papers. It has previously been acknowledged that insufficient drying impacts on the sample elution time which is likely to contribute to the loss in detection seen here. On visual inspection following blood spot elution, filter papers stored under humid conditions remained red, suggesting incomplete recovery of the blood-product [38]. Exposure to heat and moisture has been suggested to cause damage or denaturation of RNA not only by the formation of nicks in the nucleic acid strands, or by strand hydrolysis, but also by amplifying degradation caused by UV light exposure [39-42]. Removing moisture by overnight drying and inclusion of desiccant not only prevents physical destruction of the paper, but will also slow RNA degradation.

The general-purpose 3MM paper permitted detectable RNA recovery as often as the 903 Protein Saver Card and more often than the FTA Classic Card under all conditions. As this filter paper has no specific sample preservation properties and is generally used for DNA and serological sample collection, it was not expected to perform as well as it did. Previous success has been reported for viral RNA storage on the 3MM filter paper, with transcripts being detected after twelve weeks of storage at 32°C, without a loss in sensitivity [43]. Studies conducting RT-PCR from measles virus RNA stored on the 3MM filter paper have shown successful amplification following twenty-five weeks of storage at room temperature, but a loss in sensitivity with increasing temperature, and detection for just one week post storage for papers incubated at 37°C in humid conditions, due to fungal growth [44]. These results are promising, and use of 3MM is particularly appealing due to its low cost and common use as a substrate for DNA collection [23,45]. A recent publication provided evidence that this paper is indeed suitable for Pfs25 mRNA storage [34]. When this publication was in preparation, Pritsch *et al.* published similar research showing the suitability of 3MM for Pfs25 mRNA storage for low density gametocytes [34]. Results are in broad agreement: 3MM paper is suitable for Pfs25 mRNA storage, higher storage temperatures may lead to a lower sensitivity of gametocyte detection, and filter paper Pfs25 mRNA collection may lead to higher RNA yield than RNA extraction from whole blood samples. The manuscript by Pritsch provides additional details on the stability and quantification of Pfs25 mRNA; the current manuscript has limitations in the sense that RNA yield from filter papers was not determined. The analyses were restricted to gametocyte prevalence and some RNA degradation may therefore have remained undetected. However, the current manuscript extends previous reports on the use of filter paper for RNA storage by the concurrent examination of the sensitivity of RT-PCR, by showing a side-by-side comparison of three filter papers and by examining the effects of a large range of storage conditions including a combination of high temperature and high humidity, which is particularly relevant for field sample collection.

In addition to Pfs25 mRNA stability and recovery, the cost and operational ease are important considerations when choosing a methodology. The filter papers used in this study differ substantially in cost from USD $2 for 100 papers for the 3MM, $133 for 100 903 Protein Saver Cards, to approximately $400 for 100 of the FTA classic cards, in 2011. Whilst the extraction methods performed equally well in the small comparison conducted, the guanidine-based extraction is more affordable at $1.2 per reaction and is more amenable to large-scale extraction. The Qiagen protocol, complete with Qiashredder homogenization and column DNA digest, is more expensive at $4.5 per extraction. The individual expense of molecular detection is $7 per reaction for QT-NASBA, but amounts to ~ $14 for RT-PCR as it requires a DNA digest and a DNA control per sample, to exclude the possibility of DNA contamination. The combination of 903 Protein Saver Cards (which allows a minimum of five spots per card), guanidine extraction and QT-NASBA detection gives a total cost of $8.5per sample. The cheapest combination of methods is the 3MM paper, guanidine-based extraction and QT-NASBA for detection, which would amount to approximately $8.22 per sample.

## Conclusions

In summary, these findings indicate that filter paper cards can be used for collection and storage of Psf25 mRNA for detecting low gametocyte concentrations. The 903 Protein Saver Card and 3MM filter papers are both recommended as they appear particularly promising in this respect and appear to be robust storage media under temperatures higher than those conventionally used for RNA storage. Thorough drying of papers was more important for successful gametocyte detection than the temperature under which samples were stored.

## Competing interests

The authors declare that they have no competing interests.

## Authors’ contributions

SJ, TB, CD, CH and RH designed the experiments, RS contributed reagents for the study, MVB cultured the parasites and SJ, TA, KT and CH set up the study. SJ extracted the RNA, performed the RT-PCR and QT-NASBA and CS provided RT-PCR primers and contributed to RT-PCR protocol design. SJ, TB and CD interpreted the data, SJ, TB and PC performed the statistical analysis and SJ and TB wrote the manuscript. CD and CS revised the manuscript. All authors read and approved the final manuscript.

## Supplementary Material

Additional file 1 Table S1. Primer sequences for quantitative nucleic acid sequence based amplification (QT-NASBA) and reverse transcription polymerase chain reaction (RT-PCR). Click here ofr file

Additional file 2 **Table S2. Detection by RT-PCR of low-density gametocytes stored on filter papers under a range of incubation conditions.** Description of data: Data is represented as percent detection, and number of successes/number of tests, for gametocytes stored on Whatman 903 Protein Saver, the Whatman FTA Classic Card and the Whatman 3MM filter paper for five weeks (top value) and 13 weeks (bottom value). All negative controls were negative (data not shown), NT represents a condition that was not tested. Click here ofr file

Additional file 3 **Table S3. Detection by QT-NASBA of low-density gametocytes stored on filter papers under a range of incubation conditions.** Description of data: Data is represented as percent detection, and number of successes/number of tests, for gametocytes stored on Whatman 903 Protein Saver, the Whatman FTA Classic Card and the Whatman 3MM filter paper for five weeks (top value) and 13 weeks (bottom value). All negative controls were negative (data not shown), NT represents a condition that was not tested Click here ofr file
